# The use of patient‐reported outcome measures by healthcare professionals in specialized asthma management centers in French‐speaking Belgium: A mixed‐methods study

**DOI:** 10.1002/clt2.12248

**Published:** 2023-05-13

**Authors:** Gilles Louis, Bernard Voz, Michèle Guillaume, Delphine Kirkove, Benoit Pétré

**Affiliations:** ^1^ Department of Public Health University of Liège Liege Belgium

**Keywords:** asthma, mixed‐methods study, PROMs, secondary care

## Abstract

**Introduction:**

Recently, the literature has promoted the use of patient‐reported outcome measures (PROMs) in clinical practice as a means to foster patient engagement. However, conditions necessary to support the use of PROMs to encourage asthma patient engagement are not clearly defined. Therefore, we sought (1) to explore the current and ideal use of PROMs by healthcare professionals (HP) in specialized asthma management centers in French‐speaking Belgium and (2) to understand under which conditions the use of PROMs contributes to patient engagement.

**Methods:**

We undertook a mixed‐methods study with both anonymous online survey and in‐person qualitative semi‐structured interviews conducted with HPs to understand their perspectives on the routine use of PROMs. HPs were recruited from 16 asthma centers (French‐speaking Belgium) identified via the Belgian Respiratory Society.

**Results:**

Of the 170 HPs identified from the 16 participating centers, 51 (30%) responded to the survey (*n* = 51) and 11 completed semi‐structured interviews. 53% (27/51) of the surveyed HPs reported using PROMs primarily for asthma monitoring and clinical research while all reported that PROMs should primarily be used in practice to facilitate communication with the patient and to address neglected aspects of the care relationship such as the psychosocial aspects of the disease. The qualitative interviews revealed avenues for moving from a medical‐centered and utilitarian use of PROMs to a use serving patient engagement. This would require HPs to go beyond their current representation of PROMs, to use instruments offering a more holistic image of the patient, to incorporate PROMs into a digital tool and to integrate PROMs in a patient education process.

**Conclusion:**

The main findings of this study suggest relevant avenues for using PROMs in ways that support patient engagement.

## 1 INTRODUCTION

Asthma is an important public health problem affecting approximately 334 million people worldwide^1^ and 43 million people in Europe.^2^ In addition to its high prevalence, asthma is also responsible for major direct (e.g., hospital admission and the cost of pharmaceutical medicines) and indirect (e.g., school and work days lost because of exacerbations) economic costs^3^, ^4^ and has a negative impact on asthma patients' quality of life.^5^, ^6^ To deal with the public health implications of asthma, several studies consider patient‐centered care as a lever to contribute to the improvement of patients' health and the quality and safety of care.^7^, ^8^, ^9^ At the beginning of this century, the Institute of Medicine (Washington D.C., USA) recognized patient‐centered care as a key goal for improving health care systems^9^ and provides a definition according to which the patient‐centered care is “a care that respects and responds to the individual patient's preferences, needs and values and ensures that clinical decision incorporates patients' values”.^10^

Various initiatives—such as shared‐decision making,^11^ therapeutic patient education,^12^ and self‐management^13^—are part of this perspective of patient‐centered care. Next to these initiatives, the scientific literature has recently recognized the inclusion of patient‐reported outcome measures (PROMs) in clinical settings as a way to encourage patient‐centered care.^14^, ^15^, ^16^ A PROM is an instrument (e.g., questionnaire) that includes any outcome evaluated directly by the patient himself or herself and is based on patient's perception of his/her health status, disease, symptoms, health‐related quality of life (HRQL), and treatment(s).^17^ Although they also capture the patient's perspective, patient‐reported experience measures (PREMs) are different from PROMs.^14^, ^15^ Unlike PROMs, which assess how patients experience their illness and its impact on their lives, PREMs assess how patients experience their care process (e.g., satisfaction with information given by doctors or nurses).^14^, ^15^

PROMs were originally developed for use in clinical research, as secondary endpoints in clinical trials, to assess the efficacy and cost‐effectiveness of care of the treatment.^18^, ^19^ Over the last decade, PROMs have taken a new role by being increasingly collected and used in clinical practice^19^, ^20^, ^21^ to improve the detection of patient problems, to monitor changes in patient outcomes over time, to support clinical decision‐making, and to engage patients in their care. ^22^, ^23^, ^24^ Although the routine use of PROMs has gained importance in Anglo‐Saxon countries following the implementation of national programs^14^ (e.g., PROMs program launched in England in 2009), nothing similar is currently planned in Belgium. The lack of interest in PROMs in the field of asthma in Belgium raises questions even more so that international guidelines (e.g., GINA) recommend their routine use for asthma management.^25^, ^26^ Moreover, PROMs can constitute a simple and effective means of identifying patient preferences, a prerequisite for patient engagement in their care.^24^, ^27^ However, the conditions necessary to support the use of PROMs to encourage asthma patients' engagement in their care are not clearly defined in the literature, and further studies exploring the use of PROMs by healthcare professionals (HP) are essential to help researchers and policy makers gain understanding of how these tools impact clinical decision‐making.^28^

Therefore, in this study, we sought (1) to explore the current and ideal use of PROMs by HPs in specialized asthma management centers (secondary care) in French‐speaking Belgium and (2) to understand under which conditions the use of PROMs in the asthma patient‐HPs relationship contributes to improve patient engagement in their care.

## 2 METHODS

### 2.1 Study design

We conducted a mixed‐methods study with a sequential explanatory design.^29^ In this study design, the quantitative data are further explored with the qualitative data. For the quantitative study, an optional and anonymous online survey was sent to HPs from all centers specialized in asthma management in French‐speaking Belgium (*n* = 19). These centers were identified via the severe asthma registry of the Belgian Respiratory Society (BeRS). For the qualitative study, the survey data were used to construct an interview guide. The latter was used to conduct semi‐structured interviews with HPs who agreed to participate in this part of the study. These interviews were conducted to gain an in‐depth understanding of HPs' perspectives. This study was approved by the Liege University Hospital Ethics Committee (Protocol number: 2021/106).

### 2.2 Online survey

#### 2.2.1 Participants and data collection

The target population was any HPs (pneumologist, nurse, physiotherapist, psychologist, or social worker) caring for adult asthma patients and working in a specialized asthma management center identified through the severe asthma registry of the Belgian Respiratory Society. The registry includes 19 specialized asthma management centers in French‐speaking Belgium. Each center was integrated in a type of hospital structure, divided as follows: university hospital (*n* = 3), hospital with a university character (these are general hospitals that have been assigned a minority of beds managed by university authorities, without missions of teaching and research) (*n* = 5) and classic general hospital (*n* = 11).

Participants were recruited in two main steps. First, the principal investigator of the current study (GL) sent an email to all identified center heads asking if they would agree to participate in the self‐administered survey (a questionnaire) made available through an online platform. Second, center heads who responded positively disseminated the online survey link to all their HPs involved in asthma care. The survey invitation stated that it was optional and that results would be anonymous. To collect as many data as possible, the principal investigator sent reminder emails on three occasions to all center heads who agreed to participate in the survey. The online survey link was accessible between June 2021 and October 2021.

In addition to collecting socio‐demographic data the survey was composed of three main parts constructed from relevant scientific literature.^15^, ^27^, ^30^, ^31^ The first one explored the vision that HPs have of patient‐centered care through 7 questions scored on a 10‐points Likert scale (1 means not at all agree, while 10 means strongly agree). It assessed, on the one hand, HPs' attitudes about the care relationship, and on the other hand, the care relationship as it is really in the practice of HPs. The first part of the survey was accessible to all participants whether or not they use PROMs routinely. The second and third parts of the survey explored the current and ideal use of a medium (PROMs) in the HPs‐patient relationship.^15^, ^23^, ^24^ In the second part, HPs were asked to answer multiple‐choice questions. Apart from the first question asking whether or not participants used PROMs routinely, all other questions in the second part were restricted to participants using PROMs routinely. The third part of the questionnaire was available for all participants and explored HPs' preferences for the routine use of PROMs through multiple‐choice questions.

#### 2.2.2 Data analysis

Quantitative variables were summarized using median and interquartile range (P25–P75), while counts and percentage were calculated for qualitative variables. Regarding the vision that HPs have of patient‐centered care, a paired comparison using Wilcoxon test was performed to compare the attitudes versus the practices. The influence of socio‐demographic parameters on whether or not to use PROMs in routine practice was assessed by Chi^2^ tests. Similar questions asked in both the current use of PROMs (second part of the survey) and the ideal use of PROMs (third part of the survey) were analyzed using Chi^2^ tests. All statistical analyses were performed using GraphPad Prism software (version 9.4.1) at a significance level of 0.05.

### 2.3 Qualitative interviews

#### 2.3.1 Participants and data collection

All center heads who accepted to participate in the survey were contacted again to see if they would be willing to participate in the qualitative component of the study. The center heads who agreed to this part of the study provided the principal investigator with the email addresses of the HPs from their center. From this, a purposive sampling was carried out to obtain a diversity of profiles of the interviewees. To reach this diversity in the participant selection, special attention was paid to the following criteria: professional function within the center (pneumologist, nurse, physiotherapist, psychologist, or social worker), gender, professional seniority and the type of hospital structure in which the center is localized (university hospital, hospital with a university character, and classic general hospital).

Face to face semi‐structured interviews were conducted between January 2022 and July 2022. Verbal consent was obtained prior to the interviews. All the interviews were conducted by the principal investigator (GL) who had followed qualitative approach lessons during his training (Master in Sociology). All the interviews were audio‐recorded and transcribed into verbatim. No more new interview was conducted when data saturation was reached and confirmed through deliberation by a qualitative team (GL, BP, BV, and DK). This was achieved after 11 interviews. The interview guide (see online Supporting Information S1) was constructed based on results from the online survey, relevant scientific literature^15^, ^27^, ^30^, ^31^ and the three interacting poles (current situation, expected situation, and prospects for action) according to the “need analysis model” developed by E. Bourgeois.^32^

#### 2.3.2 Data analysis

A thematic analysis was carried out on all anonymized 11 HPs' narratives.^33^, ^34^ From the participants' narratives, the principal investigator (GL) extracted quotations to compile them into content‐similar groups called “codes”. Then, the codes were grouped into broader groups called “themes”. Following an iterative process, the introduction of a new code involved the rereading of all the narratives to ensure that the data extraction was complete. All this work contributed to the construction of a first analysis grid (e.g., list of themes). Next, three other investigators and experts in qualitative research (BV, BP, and DK) classified a sample of 100 quotations transmitted by the principal investigator (GL). Iterative deliberation resulted in team consensus on coding discrepancies, emergent codes, amended coding description, and themes choice and organization. All this process led to a new analysis grid. The principal investigator (GL) classified all the quotations inside a theme and then summarized them to bring out the main ideas. Finally, the observations were used to construct avenues presenting suitable conditions for the use of PROMs in order to support patient engagement in their care.

## 3 RESULTS

### 3.1 Online survey

#### 3.1.1 Characteristics of the participants (*n* = 51)

Of the 19 centers identified and contacted, 2 centers from classic general hospitals refused to participate in the survey. In addition, another center from a hospital with university character did not meet the inclusion criteria of the study because it only cared for asthmatics under 18 years of age. A total of 16 centers participated in the study and were distributed as follows: classic general hospitals (9 centers), hospitals with a university character (4 centers), and university hospitals (3 centers). Of the 170 HPs from the 16 participating centers, 51 responded to the survey (*n* = 51) leading to a response rate of 30%. Most of the respondents were women (59%) and pulmonologists (59%) working in a care center integrated in a university hospital (53%) and localized in the Walloon Region (73%) (Table 1).

**TABLE 1:** Socio‐demographic characteristics of participants (*n* = 51).

		Median (IQR) / Percentage(number)	Min‐Max
Gender (female)		59% (30)	
Years of service		13 (7–20)	3–40
Profession	Pulmonologist	59% (30)	
	Nurse	27% (14)	
	Physiotherapist	8% (4)	
	Psychologist	4% (2)	
	Social worker	2% (1)	
Asthma center area	Brussels‐capital region	27% (14)	
	Walloon region	73% (37)	
Type of hospital structure in which the center is located	Classic general hospital	33% (17)	
	Hospital with a university character	14% (7)	
	University hospital	53% (27)	

#### 3.1.2 Healthcare professionals' vision of patient‐centered care (*n* = 51)

For each of the 7 questions assessing the HPs' vision of patient‐centered care, there was a statistically significant difference between the attitudes and the practices (Table 2). Between question one exploring “the time spent giving simple, clear, and complete information to the patient about health problems”, and the final questions exploring, “discussion with the patient of the ways to enable him/her to be more autonomous”, there was an increasing augmentation in the gap between attitudes and practices showing that HPs' attitudes were more supportive of patient‐centered care than their practices (Table 2).

**TABLE 2:** Comparison test between healthcare professionals' attitudes and practices (*n* = 51).

	It is my role to do this (attitude) mean (SD)	In my practice, I can do this (practice) mean (SD)
1. Take the time to give the patient clear, simple, and complete information about their health problems	9.5 (1.5)	8.9 (1.7)***
2. Take the time to ensure that the patient has understood the various information I have provided	9.6 (0.9)	8.4 (1.5) ****
3. Discuss with the patient the different choices of care and aids possible, considering his or her life project	8.4 (2.2)	6.8 (2.1)****
4. Recognize the value of the patient's experience in relation to his or her health problems and life contexts	8.4 (1.9)	6.5 (1.8)****
5. Use questionnaires to help patients share their experiences related to their health problems and life contexts	7.9 (1.5)	5.8 (2.3)****
6. Discussing with the patient the most appropriate ways to enable them to be more competent and autonomous in their care	8.2 (1.7)	6 (2.2)****
7. Encourage the involvement of family members in the patient's care at home	6.5 (1.9)	3.7 (2.4)****

*Note*: ***Significant at the *p* < 0.001 level; **** Significant at the *p* < 0.0001 level.

#### 3.1.3 The current use of PROMs in routine by healthcare professionals (*n* = 27)

A slight majority of participants (*n* = 27, 53%) reported a routine use of PROMs (Table S1). There were significant differences in whether or not PROMs were used routinely based on socio‐demographic characteristics. The proportion of participants using PROMs routinely differed significantly by occupational status (Chi^2^ test, *p* = 0.0035) and was higher among respiratory physicians than non‐physician health workers (Figure S1). Moreover, the proportion of participants using PROMs routinely differed significantly by hospital structure (Chi^2^ test, *p* = 0.0236) and was higher in university hospital and hospital with a university character than in general classic hospital (Figure S1). The proportion of participants using PROMs routinely differed significantly by gender (Chi^2^ test, *p* = 0.0054) and was higher among men than women (Figure S1). There was no statistically significant influence of HPs seniority on the use of PROMs (Mann–Whitney test, *p* = 0.14) (Figure S1). Of the 53% respondents usually using PROMs (*n* = 27), 75% and 52% declared they had knowledge of PROMs through international guidelines and scientific articles respectively (Table S1). The top 3 reasons why they currently use PROMs routinely were to monitor asthma and its progression (81%), to do clinical research (63%), and to allow patients to share their experiences related to their disease (52%) (Table S1).

Regarding the type of PROMs used, an overwhelming majority of participants (88%) responded that they use asthma‐specific PROMs. The other participants declared using generic PROMs or even both types of PROMs (Table S1). The majority of participants declared that PROMs were completed during a face‐to face consultation (52%) through paper questionnaires (77%) and that data from PROMs were analyzed only by pulmonologists (88%) (Table S1). The top three reasons given by the 47% of respondents not using PROMs (*n* = 24) were the insufficient knowledge of PROMs among health professionals, the time constraints and the lack of financial resources (Table S1).

#### 3.1.4 The ideal use of PROMs in routine by healthcare professionals (*n* = 51)

Training on the use of PROMs (80%), more time (65%) and more resources (57%) were the top 3 suggestions given by all the respondents to improve the routine use of PROMs (Table S2). As for the reasons why the PROMs should be used in routine, the top 3 responses given by all the respondents were to allow patients to share their experiences related to their disease (71%), to address neglected aspects such as the psychosocial aspects of the disease (69%), and to facilitate communication with asthma patients (67%) (Table S2).

**FIGURE 1:**
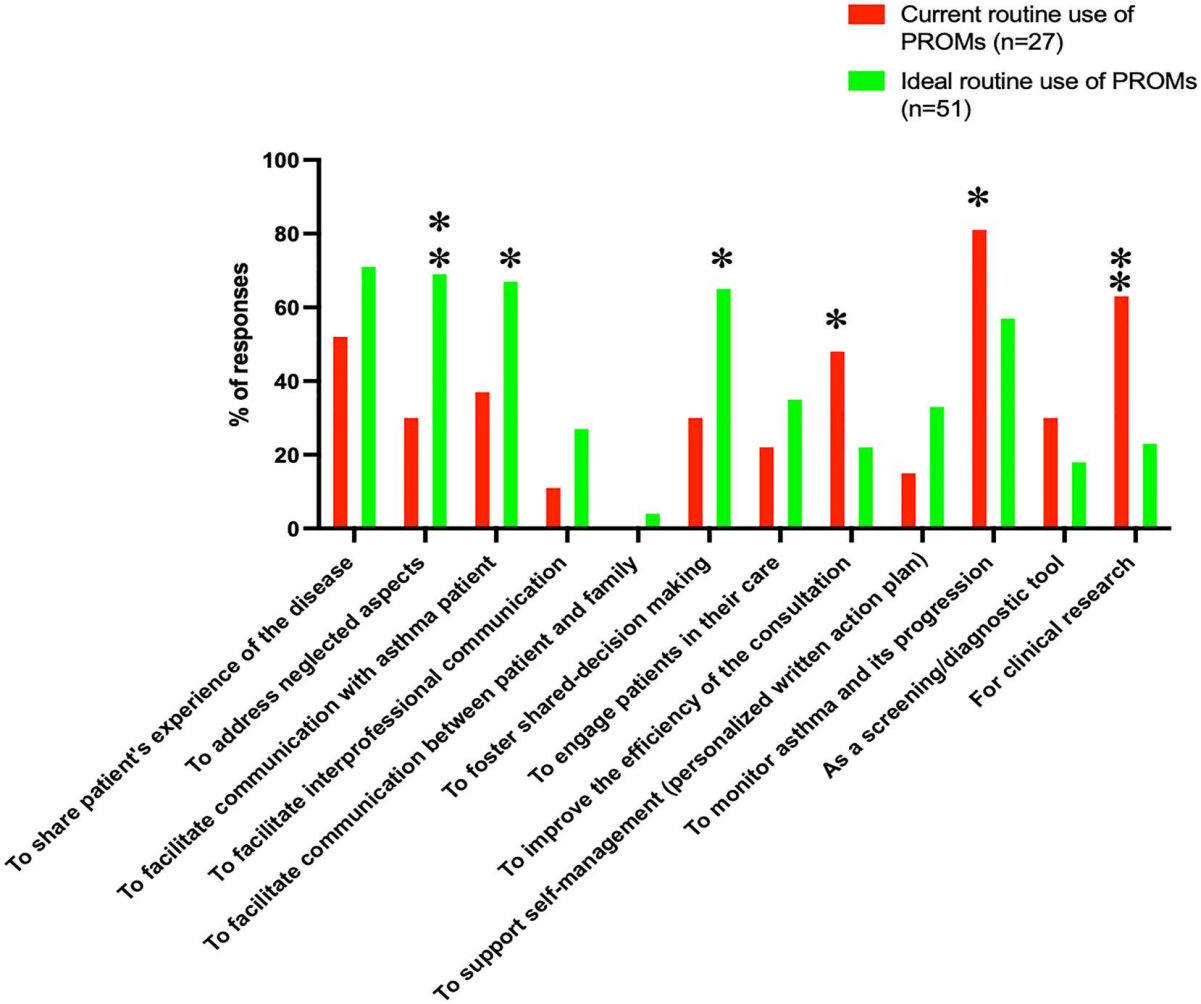
Reasons for routine use of PROMs, comparison between current use and ideal use. PROMs, patient‐reported outcome measures. * Significant at *p* < 0.05 level; ** significant at *p* < 0.01 level.

A majority of participants responded that PROMs should be completed before consultation (45% at patient home and 27% in the waiting room) through a digital medium (61% of participants with 43% via a mobile application and 18% via a digital questionnaire) (Table S2). 37% of the participants declared that data from PROMs should be analyzed and interpreted by pulmonologists while 41% of participants declared that other HPs should be involved in the analysis process (Table S2). The comparison between the reasons for the current use and ideal use of PROMs in routine is presented in Figure 1.

### 3.2 Qualitative interviews

**FIGURE 2:**
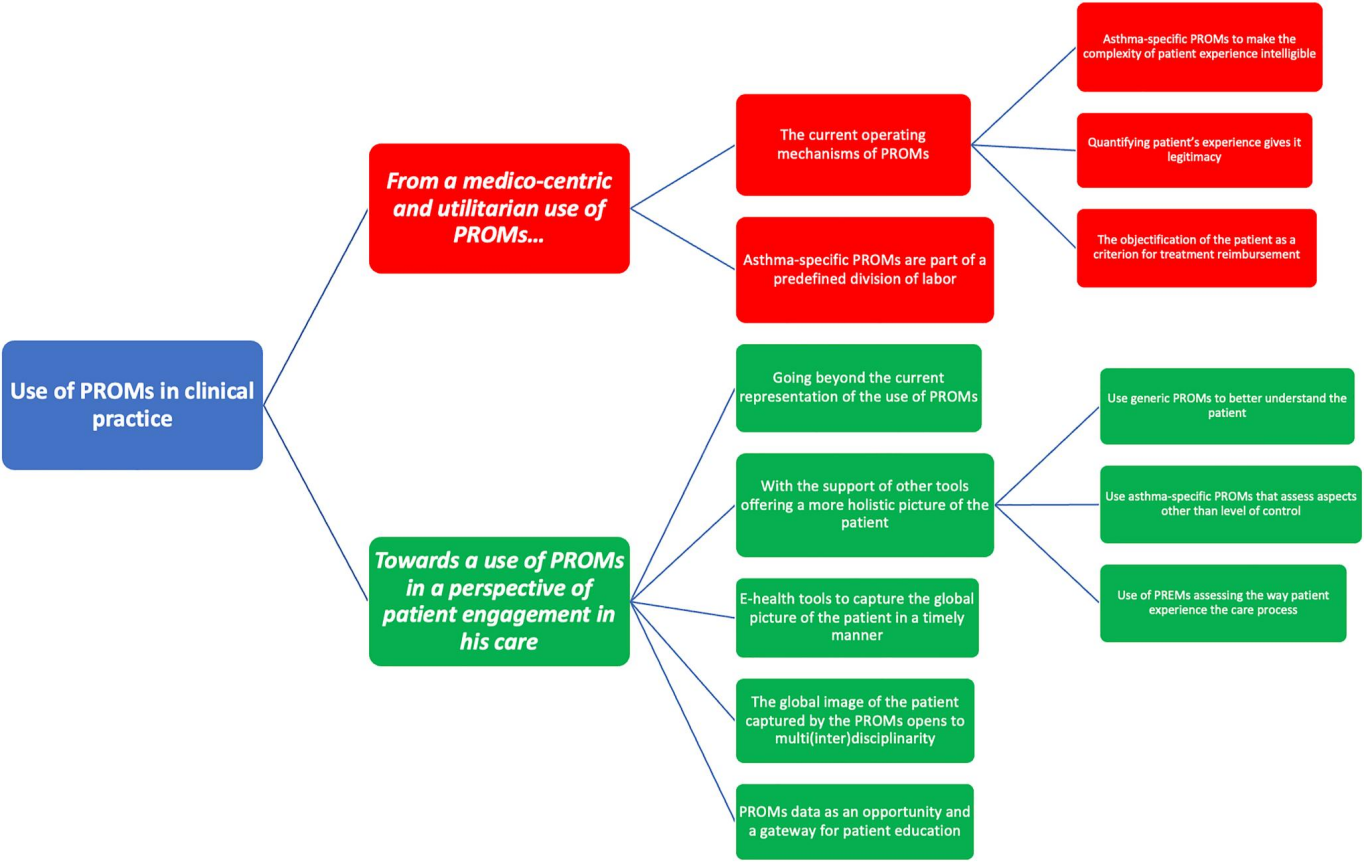
Thematic tree of the use of PROMs in clinical practice by health professionals from asthma centers. PREMs, patient‐reported experience measures; PROMs, patient‐reported outcome measures.

We conducted 11 semi‐structured interviews with HPs having different profiles and working in different types of hospitals (Table 3). The interview time ranged between 37 and 75 min. The transcribed interviews were coded and organized into two main sections, each subdivided into (sub)themes (Figure 2).

**TABLE 3:** Socio‐demographic characteristics of participants (*n* = 11).

		Percentage(number)
Gender (female)		64% (7)
Profession	Chief pulmonologist	28% (3)
	Pulmonologist	36% (4)
	Nurse	18% (2)
	Physiotherapist	9% (1)
	Psychologist	9% (1)
Asthma center area	Brussels‐capital region	27% (3)
	Walloon region	73% (8)
Type of hospital structure in which the center is located	Classic general hospital	18% (2)
	Hospital with a university character	27% (3)
	University hospital	55% (6)

#### 3.2.1 From a medico‐centric and utilitarian use of PROMs

##### The current operating mechanisms of PROMs

- *Asthma‐specific PROMs to make the complexity of patient experience intelligible*

The interviewees voiced that asthma‐specific PROMs are a way to quantify asthma patient's experience and thus to make the complexity of experience intelligible to health professionals. Asthma‐specific PROMs are as easily interpreted as bioclinical parameters.

You see, when you question a patient, you can have a whole range of answers, but the questionnaires specific to asthma make it possible to talk about a somewhat complex situation in more objective and reproducible terms.

(pulmonologist 2, hospital with a university character)

- Quantifying patient's experience gives it legitimacy

The interviewees explained that using asthma‐specific PROMs is a way to introduce the patient's perspective reliably and validly into the care relationship, thanks to the close proximity between respiratory function parameters and asthma‐specific PROM data.

I find that the asthma control test (ACT) and the asthma control questionnaire (ACQ) are very good. They allow monitoring of patients because it is really linked to the symptoms they describe in their daily life. In general, when these PROMs show significant disturbance, it hints towards an instability of asthma. Moreover, we often see a correlation between the lung function tests and results of questionnaire.

(pulmonologist 1, university hospital)

- *The objectification of the patient as a criterion for treatment reimbursement*

Asthma‐specific PROMs are tools that freeze the patient's perspective into “something” that is recognized as necessary for reimbursement of biological treatments. Reimbursement for biotherapy treatments is conditional on the collection of these “things” capturing the patient's perspective.

So to get the agreements, the patients have to be seen after 4–6 months and have check‐up. There was a time where PROMs was needed to have reimbursement of a biologic. So it was imposed by the policy. If they want to be reimbursed for their products, the patients must come to the clinic because they must prove that they still need them. So, in that context, they must complete the ACT, the ACQ and the asthma quality of life questionnaire (AQLQ). We are also kind of in those systems where they have to come in to get their reimbursement for their treatment.

(Physiotherapist, University Hospital)

##### Asthma‐specific PROMs are part of a predefined division of labor

The use of asthma‐specific PROMs does not disrupt the predefined division of labor dominated by the medical profession. The non‐physician health worker role is to accompany the patient in filling out the questionnaires, while the physician's role is to analyze and interpret the results of the PROMs.

I think that we are really there to guide the patient, so that the questionnaires are filled out in the best possible way, because we can have completely different results depending on how the patient responds, so I think that the nurse is there to guide him. The one who will decide must remain the doctor.

(nurse 2, hospital with a university character).

For me, it is logical that it should be interpreted by pulmonologists, because these questionnaires are specific to asthma, so I think that the best people to interpret them in a department specialized in asthma are pulmonologists.

(Nurse 1, university hospital)

#### 3.2.2 Toward a use of PROMs in a perspective of patient engagement in his care

##### Going beyond the current representation of the use of PROMs

Interviewees recognized the value of an alternative use of PROMs that goes beyond the medical‐centric and utilitarian use of PROMs. This alternative use of PROMs involves going beyond the score and understanding what the score means to initiate a dialogue with the patients about what really matters to them.

Clinical research plus monitoring asthma, that's already a pretty good use. Now, I think we can add to that. It goes a step further actually. It's interesting to start with PROMs results as a basis for discussion with the patient because sometimes, as I said earlier, we find aspects that would not be spontaneously discussed with the patient. From there, we can move on to something else, to sometimes redirect the patient to a psychotherapist if necessary but not systematically of course. PROMs results may be an opportunity to discover something else behind asthma itself.

(Psychologist, university hospital)

So even if we collect them, not everyone looks at them. There are “missed opportunities”. We could probably reinforce some of the messages based on those results.

(Chief pneumologist 3, hospital with a university character)

To achieve this alternative use, interviewees voiced that HPs should be made aware of the benefits of PROMs. This would especially require the scientific demonstration of the value of routinely collecting and using PROMs.

Perhaps make pulmonologists aware of the interest of PROMs. Doing what you do, a doctoral thesis by showing the interest of the various PROMs and then publishing the results of your research to show that it is important. That is, I believe, an essential step if you want things to move.

(Pulmonologist 4, general classic hospital)

##### With the support of other tools offering a more holistic picture of the patient

- *Use generic PROMs to better understand the patient*

The interviewees voiced that involving patients in their care requires more encompassing tools than asthma‐specific questionnaires. Some of them have recognized the interest of introducing, in addition to asthma‐specific PROMs, generic PROMs in order to gain in understanding the patient's globality.

(Generic PROM) It should be an opportunity to address another part of the disease, to address other areas, especially the patient's social condition, his living environment, his relationships with others, the disability that this disease can cause…

(Chief Pneumologist 1, university hospital)

- Use asthma‐specific PROMs that assess aspects other than level of control

Others believed that using asthma‐specific PROMs that assess aspects other than the level of control, such as asthma quality of life questionnaire, would contribute to a better understanding of the patients and its concerns.

It's true that maybe the AQLQ, unlike the ACT and the ACQ opens up even more, opens up enormously. There are 32 questions, it goes up to the fear of medication, as well as the weather, allergies, ability to do activities, ability to see people, chat with people. So you see, I think it's good, but unfortunately it's rarely used in consultation.

(nurse 2, hospital with a university charactsoer)

- Use of PREMs assessing the way patient experience the care process

Others believed that taking into consideration the patient's experience of the care they received can provide a more global picture of the patient.

I think that everything that is done to facilitate patient care is very positive. If the patient has problems coming to the hospital, he won't come easily. He will either go to his GP or will even be deprived from a medical visit. By contrast if everything is done so that the patient comes easily at the hospital, it is likely that he will come more easily, and he will be much more involved in the care of his pathology. So having questionnaires that assess those aspects would be great. Not focusing only on the disease but assess how the patient goes globally and try to help outside the disease field if necessary. We come back again, it's the global care of the patient and it's extraordinary.

(Nurse 1, university hospital)

##### E‐health tools to capture the global picture of the patient in a timely manner

The interviewees raised the interest of e‐health tools as a simple way to share the results of PROMs (e‐PROMS) between the patient and the physician and to focus on specific elements reported by the patient that are recognized as important by the e‐health tool, while overcoming time constraints. It can also be a way to more easily take into account (generic) PROMs data in the consultation process and expand the care of asthma patients.

Now why not also imagine an online system that people do at home or in the waiting room via a tablet or an application allowing the questionnaires to be digitally transmitted. This is what we do with clinical studies, the results are transmitted directly to a server. Then we would have access to the complete and already analyzed results of the PROMs, where we could even see the good and bad results of the PROMs graphically. This would allow that the practitioner does not waste time.

(Chief Pneumologist 2, university hospital)

##### The global image of the patient captured by the PROMs opens to multi(inter)disciplinarity

The few respondents who used asthma‐related quality of life questionnaires routinely reported that poor results in some quality‐of‐life dimensions may lead the physician to refer the patient to another health care professional. Other interviewees who were not using asthma quality of life questionnaire recognized the potential of the PROMs to foster multidisciplinarity.

So, if I see the emotional function that is pathological, I re‐interview him about his symptoms. If I see he has dizziness, paresthesia in the extremities I can also suspect hyperventilation and we refer them to the psychologist of the service. It is important to know that asthma, especially moderate to severe asthma, is often associated with increased emotionality or stress, whatever the causes or consequences. So, the AQLQ allows us to identify certain problems and therefore perhaps strengthen the link with a given patient and the link, the discussion with the other healthcare workers around the patient… that's how I see it.

(Chief pulmonologist 1, university hospital)

If at the AQLQ activity level it's having more trouble, we could redirect to the respiratory physiotherapist, for example. I think that can play that role, but unfortunately, they don't play that role right now, not here at least. But in ideal use, that would be ideal yeah (laughs).

(Pulmonologist 2, classic general hospital)

##### PROMs data as an opportunity and a gateway for patient education

Using other tools, such as generic PROMs, can contribute to patient autonomy but are not sufficient to achieve it. The interviewees voiced that something else is needed to make the patient autonomous and engaged in his care. In this regard, using PROM data can be an opportunity to develop asthma patient education.

But among the ones we use that are specific to asthma, I don't really see how they can contribute to patient autonomy. We also use the hospital anxiety and depression scale (HAD) and you see that is perhaps a little more general. This one really tells us more about the patient's general state of anxiety. It's true that it gives us information on another fact of patient’s life and it opens up our understanding of the patient even more, but it's not going to allow us to say that the patient is going to become autonomous.

(Physiotherapist, University Hospital)

They should come with their pre‐filled questionnaire, and we should have the data, the results, at the consultation. So, in fact, there should be a form of patient education. I think that if the patient comes to the consultation and is asked to fill in the questionnaire systematically, it will become routine. We would say that to each asthma patient. I think that this would make us, in the end, have a much more global vision from the first look at his test… So, I think that, yes, it's an education that the patient must have from us whether it's a doctor, a nurse, whoever. But he must be made aware of filling out this kind of questionnaire and it's our responsibility to make them aware of it from the first consultation or as soon as the diagnosis is made, because they can really help the patient to self‐manage, as long as we have taken the time to show him how to do it and how to use it.… this is what I think…

(pulmonologist 4, classic general hospital)

## 4 DISCUSSION

### 4.1 Summary

A small majority of HPs surveyed reported using routinely asthma‐specific PROMs primarily for asthma monitoring and clinical research. Other HPs not using PROMs in routine mainly justified this situation by insufficient knowledge of PROMs and lack of time. All the HPs surveyed reported that PROMs should primarily be used in clinical practice to allow the patient to share his or her experience of living with the disease, to address neglected aspects of the care relationship such as the psychosocial aspects of the disease, and to facilitate communication with the patient. The qualitative interviews helped to better understand the gap between the current medico‐centric and utilitarian use of PROMs, and the ideal use of PROMs based on the patient's engagement in his care. Achieving the use of PROMs in a perspective of patient engagement would be facilitated by overcoming the medico‐centric representation that HPs have of the use of PROMs. This would require the use of new tools—generic PROMs or PREMs—to better understand the patient's globality that should be integrated in a e‐health tool to overcome the time constraint. However, collecting and using the global image of the patient captured by these different PROMs on a routine basis is not enough to make the patient more autonomous. This could be achieved through the opportunity offered by the routine use of PROMs to develop a patient education approach.

### 4.2 Comparison with existing literature

A few studies have explored the use of PROMs in clinical practice in a mixed methods design.^35^, ^36^ Among these studies conducted in primary care settings, Turner et al.^36^ explored the current use of PROMs and the ideal use of PROMs by GPs. In this study, the authors showed that there was a similarity in the reasons for using PROMs routinely between what is currently done and what should be done. In fact, it was found that aiding clinical management, being used as a screening/diagnostic tool, and facilitating shared decision making were the top three reasons for current and ideal use. On the opposite, in our study, we found a discrepancy between current (e.g., monitoring asthma and clinical research) and ideal (e.g., sharing patient experience, address neglected aspects and facilitating communication with patient) reasons for using PROMs in clinical practice. This gap can be explained in part by the work context of our participants. Indeed, the vast majority of participants who use PROMs came from asthma centers with an academic profile that encourages clinical research and therefore a medico‐centric and utilitarian use of PROMs.

This medico‐centric and utilitarian use of PROMs is understood, in the participants' discourse, by the place that asthma‐specific PROMs occupy in the current HPs‐patient relationship. Rather than being seen as a tool to activate a dialogue, it represents an object that quantifies the patient's experience, helping to make the complexity of the patient perspective intelligible and legitimate in the eyes of the clinicians. In this sense, PROMs are not different from a bioclinical parameter. It is in line with some previous studies,^37^, ^38^ and more especially with the recent systematic review of the literature conducted by Campbell et al.^39^ which shows that the use of disease specific PROMs leads to reduce the complexity of a condition to a numerical score and that it, therefore, provides only a piece of the patient picture.

Besides this utilitarian use of PROMs, the survey revealed another use of PROMs supporting asthma patient engagement in his care, whose understanding of the conditions of this use was provided by the qualitative interviews. While various studies demonstrated the potential of the PROMs to foster patient autonomy and patient engagement,^15^, ^16^, ^19^, ^23^, ^24^, ^40^ none demonstrated clearly how the PROMs can contribute in a practical way to patient engagement. In this study, several avenues emerged to understand how the use of PROMs in clinical practice might support asthma patient engagement in his care. A first way to reinforce patient engagement would require the HPs to go beyond their current representation of the PROMs being seen as a numerical score. This is in keeping with an editorial written by Neale and Strang,^41^ where they demonstrated that the utility of these quantitative measures is enhanced if the HPs supplement the numeric value with a more in‐depth commentary. A second way would require the use of new tools in clinical practice such as generic PROMs, PREMs and e‐PROMs. While asthma specific‐PROMs are useful for monitoring disease progression, participants consider that they should be complemented by generic PROMs assessing different aspects of quality of life (e.g., emotional, social and environmental), as well as PREMs, in order to help the clinician to better understand patient's globality. This is in line with a qualitative study conducted by Wheat et al.^42^ where the authors demonstrated that taking into consideration PROMs covering issues related to quality of life and PREMs are essential for holistic care. Nevertheless, participants considered the implementation of these additional tools to be difficult due to time constraints. To overcome this difficulty, some participants see the integration of the different PROMs into a digital tool as a solution. In this view, several studies have demonstrated the benefits of using electronic PROMs (e‐PROMs) to challenge administrative time affecting workflow, while increasing quality of care.^21^, ^43^, ^44^, ^45^, ^46^ Although having positive effects, our results suggest that these tools are not sufficient to enable patients to become more autonomous.

Consequently, our results show that (e)PROMs can be a gateway to a patient education process, the value of which has been widely demonstrated in the literature in terms of patient empowerment.^47^, ^48^, ^49^ A systematic review investigating the effectiveness of routine PROM collection in cancer patients demonstrated that for complex issues (e.g., depression, social and emotional functioning), routine collection and feedback of PROM data need to be integrated with other strategies such as education, decision‐making aids, or clear management plans in order to be useful for patients.^50^ This idea is also supported in a study conducted by Santana and Feeny,^40^ where the authors developed a conceptual framework describing the potential effect of using PROMs in routine clinical care of chronically ill patients. Among the different components composing the model, one concerns the potential of PROMs to enhance patient engagement. According to the authors, this can occur in situations in which clinicians use the PROM data to discuss and educate patients.^40^ Our results feed the model by demonstrating, in the context of asthma, that the inclusion of PROMs in an educational approach would require, in particular, the use of tools allowing the capture of a more global image of the patient, as well as an overcoming of the representation that HPs have of PROMs reducing them to a simple number masking the complexity of the context of the patients' lives. Furthermore, in a country like Belgium where patient education is not formalized and institutionalized,^51^ we believe that PROMs can represent a simple and effective trigger to initiate a form of patient education which is recognized as essential in asthma management by international guidelines.^25^

### 4.3 Strengths and limitations of the study

One strength of the study is the fact that it is the first one to explore the use of PROMs by HPs in specialized asthma management centers in French‐speaking Belgium. Another strength is the fact that this study used a mixed‐methods design which allowed us to understand more deeply the results of the survey. However, the study has several limitations. One limitation is the fact that the survey response rate (30%) might be seen as low. However, it is in line with response rates that can be found in other HP surveys in Belgium.^52^ The period at which the survey was conducted corresponded to a low COVID‐19 activity in Belgium so that we do not believe that the pandemic has had a strong impact on the response rate. Another limitation is the fact that we did not survey and interview asthma patients who would have probably given another vision that would have certainly refined our current results. Indeed, having the patient's perspective would have helped to better understand the use of PROMs in a patient engagement perspective. This area should be investigated further in a new study. One more limitation is the fact that we have limited the study to the French‐speaking part of Belgium. It would be interesting to see how the HPs use PROMs in asthma specialized centers in the Dutch‐speaking part of Belgium where public health policy priorities in the prevention and health domain are different from those developed in the French part.

## 5 CONCLUSION

In conclusion, PROMs have the potential to foster asthma patient engagement in his/her care. However, this can only be achieved if HPs go beyond their current representation of the utilitarian use of PROMs. This would require the use in the consultation of new tools—e‐PROMs, generic PROMs and PREMs—that should be integrated in a patient education process and help to better understand the patient's globality.

## AUTHOR CONTRIBUTIONS

Gilles Louis, Michèle Guillaume and Benoit Pétré contributed to the conception of the study. Gilles Louis contributed to data acquisition. Gilles Louis, Benoit Pétré, Delphine Kirkove and Bernard Voz performed data analysis. Gilles Louis, Benoit Pétré, Bernard Voz, Michèle Guillaume and Delphine Kirkove drafted and critically revised the work. All authors gave final approval of the manuscript.

## CONFLICT OF INTEREST STATEMENT

The authors declare that they have no relevant conflicts of interest.

## Consent for publication

Not applicable.

6

## Supporting information

Supporting Information S1

## Data Availability

The data generated and/or analyzed during the current study are not publicly available due to the privacy of certain personal data but are available from the corresponding author on reasonable request.
