# Supplementary material for: The use of patient‐reported outcome measures by healthcare professionals in specialized asthma management centers in French‐speaking Belgium: A mixed‐methods study
Source: Clin Transl Allergy. 2023 May 13;13(5):e12248. doi: 10.1002/clt2.12248 (PMC10182759; doi:10.1002/clt2.12248)
Supplement: Supplementary file 1 — Supporting Information S1 [file CLT2-13-e12248-s001.docx]

**Supplementary Table 1 (S1): Questions related to the current use of PROMs by healthcare professionals**

| Questions | Responses n (%) |
| --- | --- |
| Do you routinely use patient-reported outcome measures (PROMs) in your center? | Responses: 51 |
| Yes | 27 (53) |
| No | 24 (47) |
| If not, what is/are the reason(s)? (Several answers possible) | Responses: 24 |
| Time constraints | 17 (71) |
| Lack of resources (financial, material, human) | 17 (71) |
| Uncertainty about the reliability of PROMs | 1 (4) |
| The absence of a legal framework related to the integration of PROMs in the management of the asthma patient | 9 (38) |
| Insufficient knowledge of PROMs among health professionals | 18 (75) |
| The plurality of PROMs | 3 (13) |
| Lack of confidence in interpreting the results of PROMs | 1 (4) |
| Patients do not like to complete questionnaires | 2 (8) |
| Don't know | 3 (13) |
| Through which channel(s) did you find out about the PROMs you use? (Several answers possible) | Responses: 27 |
| Via a training course | 1 (4) |
| Via internet | 1 (4) |
| Via a conference | 1 (4) |
| Via scientific articles | 14 (52) |
| Via actors in the health sector | 6 (22) |
| Via patients | 0 (0) |
| Via major international guidelines (e.g., GINA) | 20 (74) |
| Don’t know | 4 (15) |
| Who initiated the implementation of routine PROMs use in your center? | Responses: 27 |
| The hospital | 1 (4) |
| The physician in charge of the center | 11 (40) |
| The medical staff | 13 (48) |
| The paramedical staff | 1 (4) |
| Don’t know | 1 (4) |
| For what reason(s) do you currently use PROMs? (Several answers possible) | Responses: 27 |
| To allow patients to share their experiences related to their illness and disease | 14 (52) |
| To address neglected aspects (e.g., psychosocial dimension) | 8 (30) |
| To facilitate communication with the asthma patient | 10 (37) |
| To facilitate interprofessional communication | 3 (11) |
| To facilitate communication between the patient and his/her family | 0 (0) |
| To facilitate shared decision making | 8 (30) |
| To engage the patient in their care | 6 (22) |
| To improve the efficiency of the consultation | 13 (48) |
| To support the personalized written action plan and self-management | 4 (15) |
| To monitor asthma and its progression | 22 (81) |
| As a screening/diagnostic tool | 8 (30) |
| For clinical research | 17 (63) |
| Don’t know | 1 (4) |
| What type(s) of PROMs do you use? | Responses: 27 |
| Generic PROMs (e.g. EQ-5D, SF-36, etc.) | 1 (4) |
| Asthma-specific PROMs (e.g. AQLQ, ACT, ACQ, etc.) | 24 (88) |
| Both (generic and specific) PROMs | 1 (4) |
| Don’t know | 1 (4) |
| When and where do your patients complete the PROMs you use? | Responses: 27 |
| During the consultation: face-to-face | 14 (52) |
| Before the consultation: at home, at the request of a health professional | 3 (10) |
| Before the consultation: in a waiting room | 8 (30) |
| During the consultation: by phone | 1 (4) |
| Don’t know | 1 (4) |
| How do your patients complete the PROMs you use? | Responses: 27 |
| Via a paper questionnaire | 21 (77) |
| Via a digital questionnaire | 1 (4) |
| Via a mobile application | 1 (4) |
| Via a physician-led interview | 2 (7) |
| Via a nurse-led interview | 1 (4) |
| Don’t know | 1 (4) |
| Who analyzes and interprets the results of the PROMs you use? | Responses: 27 |
| Pneumologists | 24 (88) |
| Nurses | 0 (0) |
| Other health care professionals | 0 (0) |
| The patient | 0 (0) |
| The patient's family member(s) | 0 (0) |
| The results are analyzed and interpreted between the different health professionals | 2 (8) |
| The results are analyzed and interpreted between the physicians, the paramedical staff and the patient | 1 (4) |
| Don’t know | 0 (0) |

**Supplementary Table 2 (S2): Questions related to the ideal use of PROMs by healthcare professionals**

| Questions | Responses n (%) |
| --- | --- |
| In your opinion, which element(s) would facilitate the routine use of PROMs in your center? (Several answers possible) | Responses: 51 |
| More time | 33 (65) |
| More resources (financial, material, human) | 29 (57) |
| Training on the use of PROMs | 41 (80) |
| The existence of a legal framework related to the integration of PROMs in the management of the asthma patient | 21 (41) |
| Don't know | 8 (16) |
| In your opinion, in which area(s) do PROMs bring the most benefits? (Several answers possible) | Responses: 51 |
| To allow patients to share their experiences related to their illness and disease | 36 (71) |
| To address neglected aspects (e.g., psychosocial dimension) | 35 (69) |
| To facilitate communication with the asthma patient | 34 (67) |
| To facilitate interprofessional communication | 14 (27) |
| To facilitate communication between the patient and his/her family | 2 (4) |
| To facilitate shared decision making | 33 (65) |
| To engage the patient in their care | 18 (35) |
| To improve the efficiency of the consultation | 11 (22) |
| To support the personalized written action plan and self-management | 17 (33) |
| To monitor asthma and its progression | 29 (57) |
| As a screening/diagnostic tool | 9 (18) |
| For clinical research | 12 (23) |
| Don’t know | 7 (14) |
| When and where would you prefer patients complete PROMs? | Responses: 51 |
| During the consultation: face-to-face | 6 (12) |
| Before the consultation: at home, at the request of a health professional | 23 (45) |
| Before the consultation: in a waiting room | 14 (27) |
| During the consultation: by phone | 0 (0) |
| Don’t know | 8 (16) |
| How would you prefer patients complete PROMs? | Responses: 51 |
| Via a paper questionnaire | 8 (16) |
| Via a digital questionnaire | 9 (18) |
| Via a mobile application | 22 (43) |
| Via a physician-led interview | 1 (2) |
| Via a nurse-led interview | 1 (2) |
| Don’t know | 10 (19) |
| Who should analyze and interpret the results of the PROMs? | Responses: 51 |
| Pneumologists | 19 (37) |
| Nurses | 2 (4) |
| Other health care professionals | 0 (0) |
| The patient | 1 (2) |
| The patient's family member(s) | 0 (0) |
| The results are analyzed and interpreted between the different health professionals | 11 (22) |
| The results are analyzed and interpreted between the physicians, the paramedical staff and the patient | 10 (19) |
| Don’t know | 8 (16) |

**Supplementary Figure 1 (S1): Comparison of the use or not of PROMs in routine by health professionals according to their sociodemographic characteristics**

**Interview guide: Routine use of PROMs in asthma**

Gilles Louis, Michèle Guillaume & Benoit Pétré.

1. Introduction

Acknowledgments + request for agreement to record the interview + anonymization.

- I would like to ask you about your representation of the use made of PROMs by health professionals in second-line care centers specialized in the management of asthma in French-speaking Belgium.
- **PROM**? It is a measuring instrument, most often a questionnaire, from which the patient is asked to report a range of information about his perception of his health, his illness, his symptoms, his health-related quality of life and satisfaction with treatment => **generic** **PROM** >< **specific** **PROM**
- What is your background and what function do you exercise within the centre/service?
- How familiar are you with PROMs?

1. From using PROMs for medical care to using PROMs for patient engagement

- How do you position yourself in relation to the summary of the results of the survey on the use of PROMs that I sent you prior to the interview?

*Follow-up points: What do these results mean to you? How do you understand these results? Do they surprise you? In what?*

- The results of the survey show **a very medical view of the use that is currently made of PROMs** by the health professionals of the centers (e.g. mainly used to monitor asthma and its evolution, as well as for clinical research; analyzed and interpreted almost exclusively by the centre's pulmonologists; mainly use of asthma-specific PROMs). How do you position yourself in relation to these results?

*Follow-up points: What do these results mean to you? How do you understand these results? Do they surprise you? In what?*

*Using PROMs to monitor asthma and its evolution, what does it mean for you? In your opinion, what type of PROMs should be used to monitor asthma and its evolution?*

*What are your fears regarding the use made of PROMs in the current situation?*

- The results of the survey show a **strong patient-centered view of the ideal use that should be made of PROMs** by the health professionals (e.g. should allow the patient to share his experience of living with the disease, address neglected aspects (psychosocial dimension), promote communication with the patient, support shared decision-making). How do you position yourself in relation to these results?

*Follow-up points: What do these results mean to you? How do you understand these results? Do they surprise you? In what?*

- *Allowing the patient to share his experience of living with the disease, what does that mean for you? How can PROMs fulfill this role? What type of PROM should be used for this role?*
- *Addressing neglected aspects of the care relationship, what does this mean for you? How can PROMs fulfill this role? What type of PROM should be used for this role?*
- *Fostering communication with the patient, what does that mean for you? How can PROMs fulfill this role? What type of PROM should be used for this role?*
- *Promoting interprofessional communication, what does that mean for you? How can PROMs fulfill this role? What type of PROM should be used for this role?*
- *Supporting shared decision-making, what does it mean to you? How can PROMs fulfill this role? What type of PROM should be used for this role?*

*Concretely, by whom should the results from the PROMs be analyzed and interpreted? How should this be done?*

- In your opinion, **what are the ways to achieve this ideal use of PROMs** (ideal situation) ?

*Dunning points :*

1. *First of all, answer this question as if you were in an ideal world without constraints? What would be the ideal solution(s)?*
2. *Next, what are the various current constraints (at the level of the caregiver/patient relationship, at the level of the centre/service or institution and at the level of health policies) that prevent the implementation of the ideal solution(s)? Are some constraints removable? How can they be overcome ?*
3. *Finally, what are the various resources currently available (at the level of the caregiver/patient relationship, at the level of the centre/service or institution and at the level of health policies) that could facilitate the implementation of the ideal solution(s)? Are some resources more relevant than others? How can digital tools (mobile application with E-PROMs) constitute a resource?*

**Representation of the expected situation**:

Desired use of PROMs that helps to guide the care relationship in partnership care perspectives

**Representation of action perspectives**:

Solutions envisaged (e.g. use of digital technology, training of health professionals, legal framework)

**Representation of the current situation**:

Very medical view of the care relationship and the use of PROMs
